# Variations in terminal branches of the popliteal artery: cadaveric study

**DOI:** 10.1007/s00276-019-02262-3

**Published:** 2019-05-27

**Authors:** Łukasz Olewnik, Piotr Łabętowicz, Michał Podgórski, Michał Polguj, Kacper Ruzik, Mirosław Topol

**Affiliations:** 1 Department of Normal and Clinical Anatomy, Interfaculty Chair of Anatomy and Histology, Medical University of Lodz, Lodz, Poland; 2 Department of Diagnostic Imaging Lodz, Polish Mother’s Memorial Hospital Research Institute, Lodz, Poland; 3 Department of Angiology, Interfaculty Chair of Anatomy and Histology, Medical University of Lodz, Lodz, Poland

**Keywords:** The popliteal artery, The anterior tibial artery, The fibular artery, The posterior tibial artery, The tibial-fibular trunk, New classification

## Abstract

**Introduction:**

Identifying the branching pattern of the popliteal artery (PA) is a vital step in planning radiological and surgical procedures involving the anterior and posterior tibial and fibular arteries. The aim of this study was to characterize the course and morphology of the terminal branches of the PA.

**Materials and methods:**

The anatomical variations in the branching patterns of the anterior and posterior tibial and fibular arteries were examined in 100 lower limbs fixed in a 10% formalin solution. A dissection of the popliteal region of the leg was carried out according to a pre-established protocol, using traditional techniques. Morphometric measurements were then obtained twice by two researchers.

**Results:**

In most cases (72%) the PA divides to form the anterior tibial artery (ATA) and a common junction for the posterior tibial and fibular arteries (type I), which further splits into the fibular artery and the posterior tibial artery (PTA). This type was subdivided into two subgroups according to whether the ATA (subgroup a) or the common junction of the posterior tibial and fibular arteries (subgroup b) had the larger diameter. Other identified variations included division of the PA into the ATA and PTA—8% (type II), trifurcation—12% (type III), the division of the PTA into the ATA and FA—8% (type IV), and aplasia of the PTA—8% (type IV).

**Conclusion:**

Although the typical PA branching type was observed, it can be classified further into two additional sub-types based on the diameter of the ATA and the common junction of the posterior tibial and fibular arteries.

## Introduction

Arterial variations are less common in the lower limbs than the upper limbs [2, 8, 13, 31, 36]. In addition, the variations that occur in the arterial network of the lower limbs are typically associated with the femoral artery and its main branches [23, 28, 33], with fewer variations being observed for the anterior and posterior tibial arteries and fibular arteries [5, 12, 19, 26, 37].

The popliteal artery (PA) arises as a continuation of the femoral artery after it passes through the adductor hiatus. The PA runs in the popliteal fossa, and it normally splits at the lower border of the popliteus muscle in the anterior tibial artery (ATA) and the common junction of the posterior tibial and fibular arteries. The ATA passes into the anterior chamber (extensors) of the leg, leaving the posterior chamber (flexors) between the tibia and fibula through the upper opening of the interosseous membrane. The common junction of the posterior tibial and fibular arteries, however, maintains its position in the posterior chamber and splits into the posterior tibial artery (PTA) and the fibular artery (FA). The PA provides blood not only for the knee, including the joint capsule and ligaments, but also for areas of the leg and foot [4, 21].

Surgical and radiological procedures of the knee joint are frequent; therefore, knowledge of possible anatomical variants of the PA and its branches is essential for everyday practice. This knowledge is important for orthopaedists performing total knee replacements, femoropopliteal and tibial reconstruction surgeries, or distal femur reconstruction and proximal osteotomies [6, 9, 10, 12, 30, 32]. It is also helpful for radiologists performing transluminal stent implantations, angioplasties, embolectomies, or diagnostic angiographies to be familiar with these variants [12, 15].

The aim of our study is to determine the variations of terminal branches of the PA that may be relevant for minimizing complications during diagnostic tests and surgical procedures.

## Materials and methods

Anatomical variations of the branching patterns of the ATA, PTA and FA were evaluated in 100 lower limbs (47 female and 53 male). The cadavers were fixed in 10% formalin solution. The mean age “at death” of the cadavers was 60.7 years (35–85). The cadavers were the property of the Department following donation to the university anatomy program. Each specimen had no signs of surgical intervention in the lower leg region. All lower limbs included in the studies were also free from vascular pathology. The local bioethics commission issued consent for this study.

- Dissection protocol

The popliteal fossa was approached and popliteal fat was removed. The common fibular nerve, the tibial nerve, and the popliteal vein were identified and separated from the tibial artery. During the dissection, the ATA, the PTA and the FA morphology types were evaluated. Following this, the distance between the intercondylar line and the point of branching of the PA was measured. As intercondylar distance represents a “potential line” between the femoral and tibial condyles, it is a good, palpable topographic site. It also marks the line of the knee joint. If the common junction of the posterior tibial and fibular arteries was present, its length was also measured. In addition, the diameter at the origin site for each artery was measured. The measurements were done with an electronic calliper (Mitutoyo Corporation, Kawasaki-shi, Kanagawa, Japan). Each measurement was accurate to within 0.1 mm, and readings were done twice, by two researchers with high experience in anatomical dissection working independently. The mean value of these two measurements was used for further calculations and analysis.

### Statistical analysis

Statistical analysis was performed using Statistica 12 software (StatSoft Polska, Cracow, Poland). A *p* value less than 0.05 was considered significant. The results are presented using mean and standard deviation unless otherwise stated.

The Student’s *t* test was used to assess the association between sexes/body sides and types of arterial branching patterns. For continuous variables, normality was first checked with the Shapiro–Wilk test. As the data was not normally distributed, the non-parametric Mann–Whitney test and Wilcoxon signed-rank test were used to compare anthropometric measurements between sexes and body sides, respectively. The Kruskal–Wallis ANOVA test, along with a dedicated post hoc test, was used to compare the measurements taken from different types of the arterial tree.

## Results

**Fig. 1:**
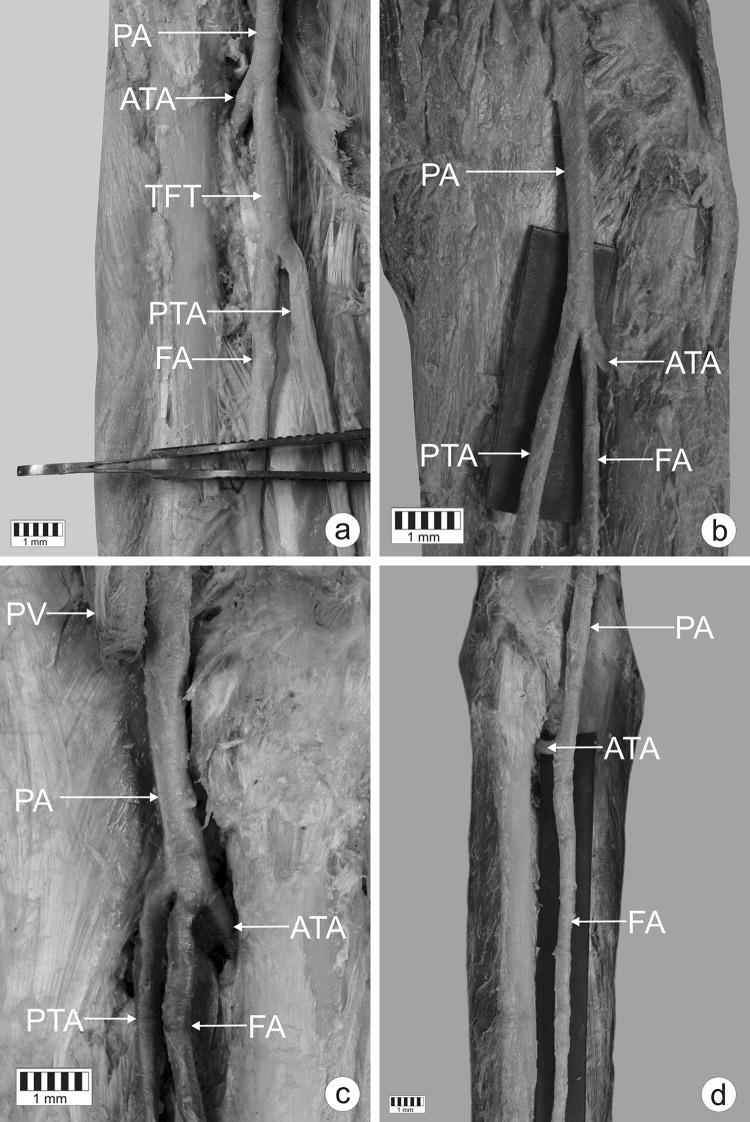
Patterns of the terminal branches of the popliteal artery. **a** Type I of terminal branches of the popliteal artery. Posterior view of the left leg. *PA* popliteal artery, *ATA* anterior tibial artery, *TFT* common junction of the posterior tibial and fibular tibial arteries, *PTA* posterior tibial artery, *FA* fibular artery. **b** Type II of the terminal branches of the popliteal artery. Posterior view of the right leg. *PA* popliteal artery, *ATA* anterior tibial artery, *PTA* posterior tibial artery, *FA* fibular artery. **c** Type III of the terminal branches of the popliteal artery. Posterior view of the right leg. *PV* popliteal vein, *PA* popliteal artery, *ATA* anterior tibial artery, *PTA* posterior tibial artery, *FA* fibular artery. **d** Type IV of terminal branches of the popliteal artery—aplasia posterior tibial artery. Posterior view of the left leg. *PA* popliteal artery, *ATA* anterior tibial artery, *FA* fibular artery

**Fig. 2:**
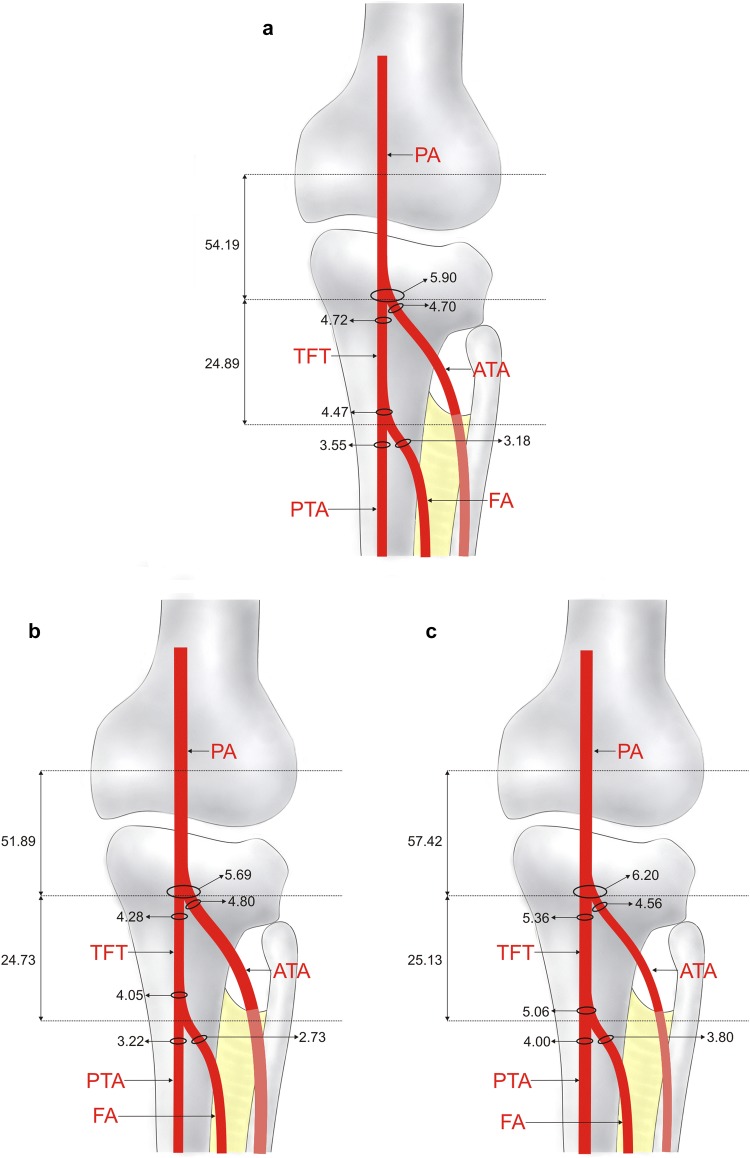
Schema of type I and its sub-types. Posterior view of the right leg. **a** Type I of terminal branches of the popliteal artery. *PA* popliteal artery, *ATA* anterior tibial artery, *TFT* common junction of posterior tibial and fibular arteries, *PTA* posterior tibial artery, *FA* fibular artery. **b** Sub-type A of the type I of the popliteal artery. *PA* popliteal artery, *ATA* anterior tibial artery, *TFT* common junction of posterior tibial and fibular arteries, *PTA* posterior tibial artery, *FA* fibular artery. **c** Sub-type B of the type I of the popliteal artery. *PA* popliteal artery, *ATA* anterior tibial artery, *TFT* common junction of the posterior tibial and fibular arteries, *PTA* posterior tibial artery, *FA* fibular artery
- Type II (Figs. 1b, 3a). The PA divides into the ATA and PTA, and then the ATA artery gives rise to the FA. Type II was present in eight cases (8%).

Four branching patterns of arteries were identified in the popliteal region:

- Type I (Figs. 1a, 2a). The PA divides into the ATA and the common junction of the posterior tibial and fibular arteries; the latter then splits into the FA and the PTA. This was the most common type, observed in 72 cadavers (72%). This type was subdivided into sub-type a (in which the ATA had a larger diameter, 42 cases—Fig. 2b) and sub-type b (in which the common junction of the posterior tibial and fibular arteries had a larger diameter, 30 cases—Fig. 2c). In sub-type b, the diameters of the PTA and FA were significantly larger than in sub-type a: 4.00 ± 1.02 vs. 3.22 ± 0.84 (*p* = 0.0001) for the PTA and 3.80 ± 1.19 vs. 2.73 ± 0.82 (*p* = 0.0001) for the FA.

**Fig. 3:**
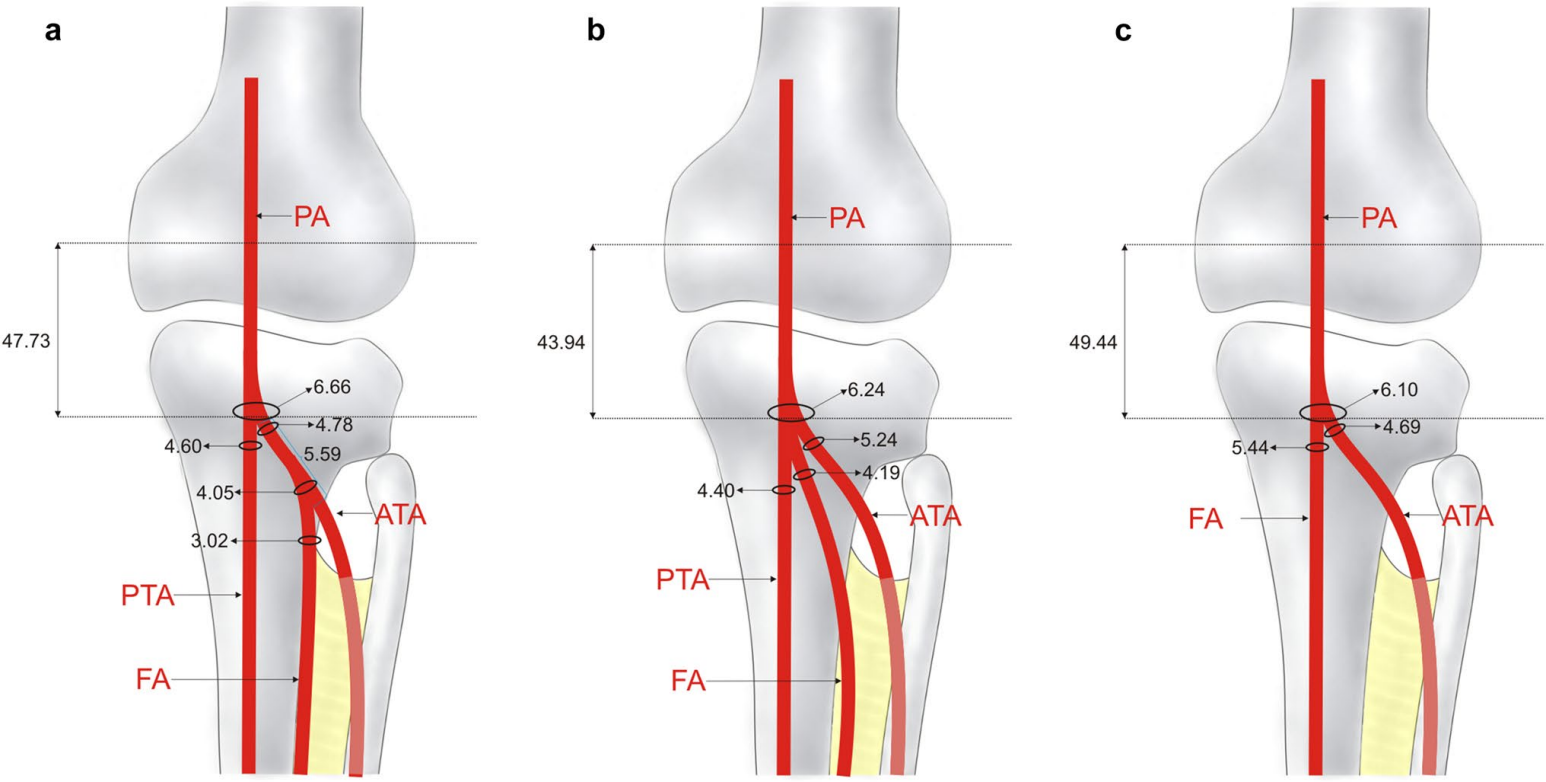
Schema of type II, III, IV of the terminal branches of the popliteal artery. Posterior view of the right leg. **a** Type II of the terminal branches of the popliteal artery. *PA* popliteal artery, *ATA* anterior tibial artery, *PTA* posterior tibial artery, *FA* fibular artery. **b** Type III of the terminal branches of the popliteal artery. *PA* popliteal artery, *ATA* anterior tibial artery, *PTA* posterior tibial artery, *FA* fibular artery. **c** Type IV of terminal branches of the popliteal artery—aplasia posterior tibial artery. *PA* popliteal artery, *ATA* anterior tibial artery, *FA* fibular artery
- Type III (Figs. 1c, 3b). The PA trifurcates into the ATA, PTA, and FA. Type III was visualized in 12 specimens (12%).
- Type IV (Figs. 1d, 3c). The PA divides into the ATA and FA. The PTA is aplastic. This type was seen in eight specimens (8%).

No difference was observed in the distribution of branching patterns between genders (*p* = 0.2128) or body sides (*p* = 0.4107). Morphometric measurements did not differ significantly between body sides, but significant differences were observed between genders (Table 1). With regard to the type of branching pattern, the diameter of the PTA was significantly smaller in type I than in all other types. Additionally, the diameter of the FA was significantly smaller in type I than in type III. Detailed morphometric data regarding the branching patterns are given in Figs. 2a–c and 3a–c. A comparison of the frequency of occurrence of types I, II and III between sexes and body sides is presented in Table 2, while a comparison of morphological parameters with regard to types of branching pattern is presented in Table 3.

**Table 1:** Differences in morphometric measurements between genders [mm]

	Females (*n* = 47)	Males (*n* = 53)	*p* value	Right (*n* = 54)	Left (*n* = 46)	*p* value
Popliteal artery diameter	5.63 (0.85)	6.37 (1.18)	0.01	6.10 (1.05)	5.93 (1.16)	0.45
Anterior tibial artery diameter	4.53 (0.83)	4.98 (0.98)	0.04	4.85 (0.97)	4.68 (0.90)	0.36
Posterior tibial artery diameter	3.84 (1.08)	3.92 (1.11)	0.76	3.99 (1.20)	3.76 (0.95)	0.30
Common junction of anterior and posterior tibial arteries with fibular artery origin diameter	4.40 (0.73)	4.96 (1.12)	0.04	4.83 (1.10)	4.63 (0.92)	0.41
Common junction of anterior and posterior tibial arteries with fibular artery end diameter	4.13 (0.73)	4.71 (1.09)	0.02	4.53 (1.07)	4.41 (0.92)	0.62
Common junction of anterior and posterior tibial arteries with fibular artery length	24.23 (13.68)	25.37 (10.97)	0.55	25.42 (10.87)	24.37 (13.34)	0.72
Fibular artery diameter	3.00 (0.87)	3.54 (1.28)	0.05	3.47 (1.17)	3.10 (1.08)	0.12
Intercondylar line—origin of the anterior tibial artery	49.71 (11.60)	54.15 (14.57)	0.01	53.09 (12.08)	50.86 (14.80)	0.41
From the origin of the anterior tibial artery to the origin of the fibular artery	4.92 (0.76)	6.09 (3.15)	0.86	6.94 (3.04)	4.58 (1.33)	0.21
Anterior tibial artery diameter at the origin of the fibular artery	3.80 (0.80)	4.23 (1.04)	0.60	4.51 (0.82)	3.70 (0.89)	0.28

**Table 2:** Distribution of branching pattern types between genders and body sides

	Females (*n* = 47) [*n* (%)]	Males (*n* = 53) [*n* (%)]	Right (*n* = 54) [*n* (%)]	Left (*n* = 46) [*n* (%)]
Type I	30 (63.8)	42 (79.3)	36 (66.7)	36 (78.3)
Type II	4 (8.5)	4 (7.6)	4 (7.4)	4 (8.7)
Type III	7 (14.9)	5 (9.4)	9 (16.7)	3 (6.5)
Type IV	6 (12.8)	2 (3.8)	5 (9.3)	3 (6.5)
*p* value	0.25		0.39

**Table 3:** Comparison of morphological parameters between types of the branching pattern

	Type I (*n* = 72)	Type II (*n* = 8)	Type III (*n* = 12)	Type IV (*n* = 8)	*p* value
Popliteal artery diameter	5.90 (1.15)	6.66 (0.97)	6.24 (1.06)	6.10 (0.59)	0.23
Anterior tibial artery diameter	4.70 (0.92)	4.78 (0.74)	5.24 (0.84)	4.69 (1.30)	0.27
Posterior tibial artery diameter	3.55 (0.99)	4.60 (0.61)	4.40 (0.75)	5.44 (0.81)	<0.01
Common junction of anterior and posterior tibial arteries with fibular artery origin diameter	4.73 (1.01)				–
Common junction of anterior and posterior tibial arteries with fibular artery end diameter	4.47 (0.99)				–
Common junction of anterior and posterior tibial arteries with fibular artery length	24.90 (12.09)				–
Fibular artery diameter	3.18 (1.12)	3.02 (0.51)	4.19 (1.22)		1.00
Intercondylar line—origin of the anterior tibial artery	54.19 (12.21)	47.73 (11.59)	43.94 (19.27)	49.44 (10.60)	0.12
From the origin of the anterior tibial artery to the origin of the fibular artery		5.59 (2.36)			–
Anterior tibial artery diameter at the origin of the fibular artery		4.05 (0.90)			–

## Discussion

To understand the arterial variations of the terminal branches of the PA, it is necessary to understand the arterial embryology of the lower limb. Most variation results from non-standard arterial division, aplasia, or hypoplasia. The sciatic artery is a branch of the internal iliac artery and is responsible for supplying blood to the early bundle of the lower limb. In the 8th week of fetal development, the femoral artery, as a branch of the external iliac artery, connects to the sciatic artery and becomes the main vessel supplying blood to the lower limbs. The sciatic artery regresses proximally and becomes the inferior gluteal artery in adults. The primitive middle and distal segments of the sciatic artery persist to form the definitive popliteal and fibularis arteries. The ATA arises as a branch of the PA, while the PTA is formed by an anastomosis between the early distal femoral artery and the PA. Complete vascularisation of the lower limb is established by the end of the 12th week of pregnancy [19]. The most common morphological variations are associated with high or normal origin, and also concern types [1, 3, 5, 7, 11, 12, 14, 16–20, 22, 24, 25, 27, 29, 34, 35, 37]. High levels of branching of the PA result from variations in the type of regression of the medial division of the sciatic artery [29]. However, when the anterior sciatic artery bundle fails to regress, a common trunk for the ATA and FA is formed, and when the distal portion of the sciatic artery fails to regress, trifurcation occurs [29].

Morphological variations of the PA occurred in 28% of cadavers in this study, which was higher than in other studies, in which the prevalence of variations ranged from 4 to 17.6% [1, 3, 5, 7, 11, 12, 14, 16–20, 22, 24, 25, 27, 29, 34, 35, 37]. A few classifications have been proposed for these morphological variants [16, 18]. A currently used classification by Kim et al. [16], divides the branching pattern of the PA into three types (I–III), with each type classified further into three sub-types (A–C) (Table 4). Type III is a novelty proposed by Kim et al. [16]; it is characterized by a PA with a normal branching pattern and sequence, but with aplastic or hypoplastic proximal portions of the ATA and/or PTA. The latest systematization of the Kim et al. classification was proposed by Demirtaş et al. [12], who propose the addition of type ID, characterized by a very long common junction of the posterior tibial and fibular arteries (110 mm), and type IIID, characterized by a hypoplastic/aplastic PTA. Our present findings indicate the need for the addition of a further subdivision for type IA based on the relative diameters of the ATA and the common junction of the posterior tibial and fibular arteries: more specifically, sub-type A1, characterized by the ATA being wider than the common junction of the posterior tibial and fibular arteries, and sub-type A2, by the common junction of the posterior tibial and fibular arteries being wider than the ATA. Although no type II branching patterns were found in any specimens, further subdivision according to vessel diameter would be advantageous.

**Table 4:** Classification modified by Kim et al. [16]

	Type I—normal division	Type II—high division	Type III—aplastic/hypoplastic
Sub-type			
A	Normal pattern	ATA arises at or above popliteus muscle	Hypoplastic—aplastic PTA
B	Trifurcation	PTA arises at or above popliteus muscle	Hypoplastic—aplastic ATA
C	Anterior tibial-fibular trunk	FA arises at or above popliteus muscle	Hypoplastic—aplastic PTA and ATA

The most common variant of the terminal branches of the PA detected by anatomical dissection is type I, with a frequency ranging from 92.5 to 97.3% [1, 14, 18, 27, 34]. This has been confirmed by angiographic studies, with the frequency of type I found to range from 88.2 to 95.8% [3, 7, 11, 12, 15, 16, 19, 20, 22, 25, 35]. Type I was also found to be the most common variant in the present study, occurring in 72% of cases (summation of patterns I, II and III).The prevalence of type II branching, according to Kim (high origin), has been found to range from 0.8 to 7.5% in anatomical studies [1, 14, 18, 24, 27, 34] and from 1.6 to 7.8% in angiographic studies [3, 7, 11, 12, 15, 16, 19, 20, 22, 25, 35]; however, this type was not observed in any of our specimens. Type III (Kim) has only been reported in angiographic studies, with an occurrence ranging from 1 to 7.6% [7, 11, 12, 15, 20, 25, 35]. Interestingly, 8% of the specimens in the present anatomical study displayed type IIIA branching according to Kim (our type IV). More precise differences in the findings of anatomical and angiographic studies are presented in Tables 5 and 6. Interestingly, the present study shows that the diameter of the PTA was significantly smaller in type I than in the other types (II, III), and the diameter of the FA in type I was significantly smaller than in type III; this is an important point for consideration when performing surgical procedures in this area.

**Table 5:** Comparison between anatomical dissection studies in popliteal artery variations (classification Kim et al. [16] extended by Dimirtas et al. [12])

Author	Types of examination	Number of lower limbs	Type IA (%)	Type IB (%)	Type IC (%)	Type ID (%)	Type IIA1 (%)	Type IIA2 (%)	Type IIB (%)	Type IIC (%)	Type IIIA (%)	Type IIIB (%)	Type IIIC (%)	Type IIID (%)
Quain [27] (1844)	Anatomical dissection	258	90.3	6.6	2.3	–	0.8	–	–	–	–	–	–	–
Adachi [1] (1928)	Anatomical dissection	770	96	0.5	0.8	–	0.9	1	0.8	–	–	–	–	–
Trotter [34] (1940)	Anatomical dissection	1168	93.4	1.3	0.5	–	1.5	2.4	1.4	–	–	–	–	–
Keen [14] (1961)	Anatomical dissection	280	90.7	0.4	4.3	–	3.6	0.4	1.1	–	–	–	–	–
Ozgur et al. [24] (2009)	Anatomical dissection	40	90	–	2.5	–	5	–	2.5	–	–	–	–	–
Olewnik (Present study)	Anatomical dissection	80	72	12	8	–	–	–	–	–	8	–	–	–

**Table 6:** Comparison between angiographic studies in popliteal artery variations (classification Kim et al. [16] extended by Dimirtas et al. [12])

Author	Types of examination	Number of lower limbs	Type IA (%)	Type IB (%)	Type IC (%)	Type ID (%)	Type IIA1 (%)	Type IIA2 (%)	Type IIB (%)	Type IIC (%)	Type IIIA (%)	Type IIIB (%)	Type IIIC (%)	Type IIID (%)
Morris [22] (1961)	Angiography	246	88.6	1.2	2.9	–	3.6	–	0.8	–	–	–	–	–
Bardsley and Staple [3] (1970)	Angiography	235	92.8	–	0.4	–	4.2	–	1.7	–	–	–	–	–
Mauro et al. [19] (1988)	Angiography	343	88	1.2	4.1	–	2.3	–	0.9	–	–	–	–	–
Kim et al. [16] (1989)	Angiography	605	92.6	1.2	2	–	3	0.7	0.8	< 0.2	–	–	–	–
Day and Orme [11] (2006)	Angiography	1037	90.7	3.2	0.3	–	4.5	–	1.1	0.2	0.8	0.1	0.1	–
Kill and Jung [15] (2009)	Angiography	1242	89.2	1.5	0.1	–	1.2	–	0.4	–	5.1	1.7	0.8	–
Mavili et al. [20] (2011)	Angiography	535	82.4	5.4	0.4	–	3.9	–	1.5	–	3.7	2.2	0.2	–
Yanik et al. [35] (2015)	Angiography	126	83.6	0.8	4.4	–	5.2	–	2.6	–	3.4	–	–	–
Calisir et al. [7] (2015)	Angiography	742	87	4.2	1	–	3.6	–	1.4	–	2.7	0.9	–	–
Oztekin et al. [25] (2015)	Angiography	495	87.5	3	1.2	–	1.4	0.4	1	–	3.3	0.6	0.4	–
Demirtas et al. [12] (2016)	Angiography	1342	88.7	2.5	0.6	0.1	2.2	0.4	0.6	–	3.5	1.2	0.1	0.1

Arterial variations in the terminal branches of the PA have great radiological, orthopedic and surgical importance, as without this precise knowledge, iatrogenic injury is possible during procedures such as vascular graft transplants, embolectomies, surgical repairs or endovascular angioplasties [12, 32]. The presence of highly branched arteries, especially the ATA, and their variability can present a number of arterial complications for such orthopedic interventions as total knee arthroplasties, high tibial osteotomies, posterior cruciate ligament reconstructions, lateral meniscal repairs and arthroscopies [17, 25]. The presence of a type III PA may be particularly problematic in this sense. In the case of a hypoplastic or aplastic PTA, the FA exhibits compensatory hyperplasia and supplies the vascular bed of the PTA with blood. In this case, the FA of the foot gives rise to the lateral plantar artery, but the medial plantar artery is usually absent [37]. The FA is also a significant vessel for the preparation of the fibular graft and gastrocnemius muscle flap [37]. In the case of possible hypoplasia or aplasia of the ATA and/or the PTA, it is particularly important to make a pre-operative determination of the adequacy of the blood supply to the foot after blocking the FA.

The main limitation of our study was the small size of the research sample. Nevertheless, its number is comparable to, or even larger than, those of samples used in similar anatomical studies [24]. The second weakness of the work is that it is based only on anatomical study; its findings would be complemented by data from angiographic studies and would improve the practicality of the prepared classification. Finally, our classification includes an analysis of vessel diameter and an evaluation of dominance. Although we are aware that postmortem changes of vessel walls can influence direct measurements, these changes should be uniform across samples and affect all vessels and are unlikely to demonstrate bias.

## Conclusion

Adequate knowledge of the high morphological variability occurring in the terminal branches of the PA is needed to safely perform correct diagnostic and surgical procedures in this region of the body. PTA diameter was significantly smaller in type I than in the other types (II, III), and FA diameter in was significantly smaller in type I than type III. Any comprehensive classification of PA branching pattern should, therefore, include two sub-types based on vessel diameter for type IA and all versions of type II.
